# Muscle energy techniques for post-stroke spasticity: mechanisms and clinical applications

**DOI:** 10.3389/fneur.2026.1773854

**Published:** 2026-04-28

**Authors:** Lifeng Qian, Yanbo Shi, Wen Liu, Qing Yao

**Affiliations:** 1 Department of Rehabilitation, Zhejiang Chinese Medical University Affiliated Jiaxing Traditional Chinese Medicine Hospital, Jiaxing, Zhejiang, China; 2 Central Laboratory of Molecular Medicine Research Center, Zhejiang Chinese Medical University Affiliated Jiaxing Traditional Chinese Medicine Hospital, Jiaxing, Zhejiang, China; 3 Jiaxing Key Laboratory of Diabetic Angiopathy Research, Jiaxing, Zhejiang, China; 4 Department of Encephalopathy, Zhejiang Chinese Medical University Affiliated Jiaxing Traditional Chinese Medicine Hospital, Jiaxing, Zhejiang, China

**Keywords:** limb dysfunction, muscle energy technique, muscle tension, pain, post-stroke spasticity

## Abstract

Spasticity is a common and disabling complication after stroke, often leading to progressive joint stiffness, restricted movement, and reduced functional independence. Current management strategies for post-stroke spasticity (PSS) are limited by inconsistent efficacy and a lack of standardized protocols. Muscle energy techniques (MET) have emerged as a promising non-invasive approach, though their mechanisms and clinical value in PSS remain poorly understood. This review summarizes available evidence on MET for PSS based on systematic searches of PubMed, Web of Science, CNKI, and WanFang up to November 2025. MET may alleviate PSS through two main routes, namely inhibiting spinal and cortical motor neuron excitability and modulating pain pathways, though the evidence for these mechanisms remains limited and comes mainly from experimental studies. Key clinical studies indicate that MET can reduce muscle tone, improve range of motion, and enhance functional outcomes, with particularly notable effects on upper limb spasticity. However, heterogeneity in treatment protocols and a shortage of high-quality trials limit the strength of current conclusions. We further discussed critical limitations, including the reliance on active patient participation, which may preclude its use in persons with stroke with significant cognitive or motor deficits. Future directions include standardizing treatment protocols and integrating MET with emerging technologies such as biofeedback and brain-computer interfaces. This review offers a mechanistic and clinical framework to support the evidence-based integration of MET into PSS rehabilitation.

## 1 Introduction

Stroke remains a leading cause of long-term disability worldwide (1). Studies have shown that the number of strokes worldwide has increased significantly over the past three decades, resulting in losses exceeding US$890 billion (2). Most persons with stroke experience degrees of functional impairment, and up to 90% develop limb spasticity during the recovery period (3). This high prevalence of post-stroke spasticity (PSS) is closely linked to greater disability, and is strongly associated with reduced mobility, persistent pain, and lower quality of life. Clinically, PSS often presents as the classic Wernicke-Mann posture, characterized by flexor hypertonia in the upper limbs and extensor hypertonia in the lower limbs (4). The condition typically emerges within 3 weeks after stroke and is marked by velocity-dependent hypertonia, hyperreflexia, and abnormal co-contraction of agonist–antagonist muscle pairs (5). Interventions that fail to address this abnormal pattern may inadvertently reinforce it, thereby impeding motor recovery. Thus, developing effective, targeted strategies to alleviate PSS remains a key priority in stroke rehabilitation.

The precise pathogenesis of PSS is still being clarified. Under normal conditions, a balance between excitation and inhibition, maintained by supraspinal control of *α* and *γ* motor neurons, keeps muscle tone within a physiological range. Stroke disrupts this balance, often resulting in relative over-activity of the γ system. A key mechanism is thought to be damage to descending corticospinal tracts, which inhibits spinal reflex circuits and leads to the velocity-dependent hypertonia and hyperreflexia characteristic of spasticity (6). This attenuation of central inhibition effectively releases primitive spinal networks, heightening the excitability of final common motor pathways (7). At the neurochemical level, spasticity may also arise from an imbalance in synaptic neurotransmission, where an excess of excitatory mediators or a deficit of inhibitory ones can precipitate or worsen symptoms (8).

Currently, non-pharmacological interventions for PSS include neurophysiological therapy, constraint-induced movement therapy, motor imagery, mirror therapy, and various physical agent modalities, often delivered through mechanical assistance or guided passive movement (9). Historically, resistance training was rarely used in PSS management due to concerns that it might worsen spasticity. However, these concerns were largely anecdotal and lacked solid empirical support. Emerging evidence suggests that appropriately dosed resistance training does not aggravate spasticity and may in fact offer meaningful rehabilitative benefits for persons with stroke (10). Despite these developments, many existing interventions share common limitations: delayed onset of measurable benefits, lack of standardization, and variable patient adherence, all of which can lead to inconsistent outcomes (11–13). These shortcomings highlight an urgent need for therapeutic options that are effective, non-invasive, and cost-efficient, specifically aiming at reducing spasticity to meaningfully improve person’s quality of life (14).

Muscle energy technique (MET) was first proposed and developed by orthopedic surgeons Fred Mitchell Sr. and Jr. in the 1950s (15). As a form of soft tissue manipulation, MET is designed to enhance musculoskeletal function and mitigate pain. The technique involves the person performing controlled isometric or isotonic contractions against a counterforce applied by the clinician (16, 17). These actions leverage neurophysiological principles of autogenic and reciprocal inhibition, with the intended outcomes of strengthening weakened muscles, reducing hypertonia, improving joint range of motion, and consequently alleviating pain by releasing tense tissues (18, 19). Emerging evidence suggests MET may offer dual benefits in PSS: reducing muscle tone and facilitating strength recovery (20, 21). However, the underlying mechanisms and optimal clinical application of MET in PSS remain incompletely understood (22).

This review aims to summarize current evidence on the mechanisms of MET in PSS, assess its clinical applications and efficacy, identify barriers to implementation, and suggest directions for future research and clinical standardization.

## 2 Method

We searched PubMed, Web of Science, China National Knowledge Infrastructure (CNKI), and Wanfang for articles published up to November 2025. Search terms included muscle energy technique, post-stroke spasticity, mechanism, and related variants. We used various combinations of the above search terms to assess the literature comprehensively. First, the relevance of the title and abstract to the content was reviewed. If they were deemed relevant, the entire paper was accessed. Studies with research focus on muscle energy technique and post-stroke spasticity were included. Other relevant additional articles were identified from similar articles listed in the search results and from references in articles obtained from the search. These additional articles were used to provide background and other supporting information.

## 3 Mechanism of MET in PSS

### 3.1 Inhibition of motor neuron excitability

The neurophysiological foundation of MET rests upon the principles of reciprocal inhibition and reflex modulation (23). The process begins when stretch applied to a spastic muscle activates intrafusal muscle spindle receptors, sending afferent signals to the spinal cord’s dorsal horn (24). This increases the excitability of *α* motor neurons in the ventral horn, triggering efferent impulses that produce a reflexive contraction (25). As this contraction raises muscle tension, it stimulates Golgi tendon organ (GTOs). GTO activation generates inhibitory feedback to the spinal cord, which dampens alpha motor neuron activity, reduces efferent drive, and ultimately promotes muscle relaxation (26). Concurrently, according to the principle of reciprocal inhibition, the central excitation of an agonist muscle is accompanied by reflexive inhibition of its antagonist. By harnessing these dual mechanisms, GTO-mediated autogenic inhibition and spinal-level reciprocal inhibition, MET can effectively normalize muscle tone, induce relaxation, and create conditions favorable for functional recovery (27). Osama et al. found that combining post-isometric relaxation (PIR) based MET with joint mobilization yielded significant immediate and short-term gains in isometric strength (28). Similarly, MET techniques employing autogenic or reciprocal inhibition have been shown to produce immediate increases in isometric strength across multiple movement planes (29).

Two key neurophysiological findings support this dual-level inhibitory effect: a prolonged cortical silent period after motor evoked potentials, and a reduced H-reflex amplitude (30, 31). The prolonged silent period reflects enhanced intracortical inhibition, suggesting stronger supraspinal suppression of excessive descending commands. The reduced H-reflex amplitude is primarily an experimental measure of spinal reflex excitability rather than a direct clinical indicator, yet it suggests a spinal effect: MET may lower *α* motor neuron excitability and thus reduce responsiveness to sensory input. In clinical settings, H-reflex latency is more commonly used to assess nerve conduction, whereas amplitude reduction provides complementary information about spinal reflex arc excitability, which is directly relevant to understanding how spasticity is reduced.

Beyond its immediate effects, MET may also promote neural plasticity that supports long-term spasticity management. The technique requires precise control over contraction direction and intensity, which generates targeted proprioceptive feedback and enhances cortical sensorimotor representation of the affected limb. This type of task-specific sensory input is thought to drive use-dependent reorganization in the motor cortex, potentially helping to form adaptive neural pathways (10). The ability to combine immediate inhibition with longer-term plasticity may explain why MET offers more sustained and fundamental benefits than passive stretching alone.

### 3.2 Modulation of pain conduction

Evidence is accumulating that MET may relieve pain, likely through a combination of manual contact and neuromuscular stimulation (32). Studies have shown it reduces pain and improves function in conditions such as sacroiliac joint dysfunction (33) and chronic non-specific low back pain (34). Meta-analyses suggest MET is a useful adjunct for pain relief, helping to improve range of motion and function (18, 35), which supports using it before other therapies to reduce nociceptive interference (36).

Promising as these findings are, the precise mechanisms by which MET relieves pain in PSS are not yet fully understood. Current hypotheses point to a combination of peripheral and central regulatory processes (37). On the peripheral side, MET may improve local circulation, reduce myofascial tension, and help deactivate trigger points through mechanical pumping that enhances lymph flow and clears algesic substances (38).

The MET addresses peripheral pain generators, notably myofascial trigger points. These hyperirritable sites are sources of local and referred pain, partly due to the sustained release of algesic substances. MET may facilitate their resolution through multiple interconnected mechanisms. The rhythmic contractions act as a mechanical pump, enhancing local circulation and lymph flow. This not only improves oxygen delivery and metabolite clearance but may also alter interstitial pressure and fibroblast activity, promoting tissue repair. Evidence suggests MET can reduce local concentrations of pro-inflammatory cytokines and desensitize peripheral nociceptors, further reducing edema and pain signaling (37). Collectively, these actions create a microenvironment conducive to fascial remodeling, collagen synthesis, and the restoration of normal tissue biomechanics, addressing both the symptoms and substrates of pain.

Neurophysiologically, MET is thought to engage endogenous pain modulatory systems. At the spinal level, this may involve the release of endogenous opioids from glial cells and interneurons, inhibiting nociceptive signal transmission. Simultaneously, at the supraspinal level, MET may influence key pain-processing regions such as the anterior cingulate cortex and insula, potentially modulating both the sensory-discriminative and affective-emotional dimensions of pain, thereby elevating pain tolerance. This enhanced supraspinal regulation likely improves sensory-motor integration and heightens cortical awareness of the affected limb, countering neglect patterns (35). Furthermore, by ameliorating the overall sensorimotor experience, MET may indirectly alleviate comorbid anxiety and depression, factors known to lower pain thresholds. The relevant evidence indicates that MET can effectively alleviate the pain symptoms of persons with sacroiliac joint pain and improve their functional status. However, the recommendation strength is level C (33), indicating that further evidence is needed.

## 4 Clinical applications for MET in PSS

The MET has been extensively studied in musculoskeletal conditions, but evidence for its use in PSS is still limited, although growing. As summarized in Table 1, only one completed randomized controlled trial has specifically examined MET for PSS to date. Yuan et al. (21) found that combining low-frequency repetitive *trans*-cranial magnetic stimulation with MET significantly improved upper limb spasticity (Modified Ashworth Scale: from 2.13 ± 1.01 to 1.00 ± 0.76, *p* < 0.05), upper limb motor function (Fugl-Meyer Assessment: from 40 to 64 points, *p* < 0.05), and activities of daily living (Modified Barthel Index: from 69 to 100 points). An ongoing trial by Yu et al. (39) is currently investigating the combination of MET with blood flow restriction training in 80 persons with stroke.

**Table 1:** Direct clinical evidence of MET in PSS.

Study	Design	Sample	Intervention	Results
Yuan et al. (21)	RCT	30 persons with PSS	MET group, or MET + low-frequency repetitive trans-cranial magnetic stimulation group	Compared with the MET treatment group, the modified Ashworth scale score of the combined treatment group significantly decreased at 8 weeks of treatment (*P* < 0.05), and the Fugl-Meyer and Modified Barthel Index scores significantly increased at 4 weeks and 8 weeks of treatment (all *p* < 0.05).
Yu et al. (39)	RCT Protocol	80 stroke persons with PSS	MET + blood flow restriction training group, or MET group	Study ongoing.

MET, muscle energy technique; PSS, post-stroke spasticity; RCT, randomized controlled trial.

Given the limited direct evidence for MET in PSS, Table 2 summarizes supportive findings from related populations and mechanistic studies. Fryer and Pearce (30) applied MET to the lumbosacral joint in asymptomatic individuals and found a significant decrease in H-reflex amplitude (*F*_1.3, 14.4_ = 13.8; *p* = 0.01) and an increase in cortical silent period duration (*F*_2, 22_ = 7.64; *p* = 0.03). These findings suggest reduced spinal reflex excitability and enhanced intracortical inhibition, shedding light on the neurophysiological mechanisms by which MET may influence motor excitability. In stroke populations, Wang et al. (20) reported that combining MET with Neurac improved upper limb motor function, activities of daily living, and pain in 100 stroke persons with diabetes and hemiplegia. In musculoskeletal conditions characterized by pain and limited range of motion, MET has shown consistent efficacy. Osama (28) and Siddiqui et al. (40) both reported significant improvements in isometric strength in persons with neck pain. Similarly, Faqih et al. (41) found that MET increased elbow flexion range of motion and reduced pain in persons with post-surgical elbow stiffness. Together, these findings suggest that MET may be effective in addressing the core impairments of PSS, including spasticity, weakness, pain, and reduced range of motion, while also highlighting the need for more direct evidence in the PSS population.

**Table 2:** Supporting evidence about PSS from related populations and mechanistic studies.

Study	Design	Sample	Intervention	Results
Fryer et al. (30)	Experiment	12 asymptomatic	MET applied to hamstrings	MET significantly increased cortical silent period duration (*F*_2,22_ = 7.64; *p* = 0.03) and decreased H-reflex amplitude (*F*_1.3,14.4_ = 13.8; *p* = 0.01). These changes point to reduced corticospinal and spinal reflex excitability. H-reflex amplitude reflects spinal *α* motor neuron excitability, and a decrease suggests lower spinal reflex gain, which is relevant to understanding spasticity mechanisms.
Wang et al. (20)	RCT	100 stroke persons with hemiplegia complicated by diabetes	MET group, Neurac group, or joint group (receives MET plus Neurac), or conventional rehabilitation group	The treatment herein achieved significant improvements in Barthel index scores, visual analog scale scores (2.71 ± 0.28), Berg Balance Scale scores, Tinetti scores, Fugl-Meyer scores, and Quality of Life scores (99.67 ± 10.62), and MET plus Neurac method obtained the best results versus both the conventional rehabilitation and monotherapy of either MET or Neurac (*p* < 0.05).
Siddiqui et al. (40)	RCT	80 persons with mechanical neck pain	autogenic inhibition (AI-MET) group, or reciprocal inhibition (RI-MET) group	The AI-MET is more beneficial than RI-MET in improving pain, range of motion, and functional disability in persons with sub-acute and chronic mechanical neck pain.
Osama (28)	RCT	78 participants with neck pain	static stretching (SS) group, or AI-MET group, or RI-MET group	Both AI-MET and RI-MET were found to be comparatively more effective than SS, however AI-MET was found to be the most effective.
Faqih et al. (41)	RCT	30 persons with post elbow fracture fixation	MET + conventional therapy group, or conventional group	MET can be used as an adjunct to the rehabilitation protocol to treat elbow stiffness and can be given safely in the early stages.

MET, muscle energy technique; PSS, post-stroke spasticity; RCT, randomized controlled trial; AI, autogenic inhibition; RI, reciprocal inhibition; SS, static stretching.

## 5 Discussion and prospects

Available evidence suggests that MET alleviates PSS through multiple mechanisms that align well with the underlying pathophysiology (34). The technique acts on the hyperexcitable *α* and *γ* motor neuron loop, helping to reduce motor neuron overactivity. By applying controlled resistance, MET can help regulate resting muscle tension. When chronically elevated, this tension contributes to ischemia, sterile inflammation, and a self-sustaining pain-dysfunction cycle (42, 43). MET may interrupt this cycle by stimulating proprioceptive feedback, normalizing aberrant discharge patterns, and reducing spinal excitability, as suggested by neurophysiological studies such as those demonstrating H-reflex modulation (15, 44). In doing so, MET supports the restoration of more coordinated movement patterns.

MET has a clear advantage over passive stretching in that it engages the patient in an active, goal-directed motor task. Passive stretching mainly targets tissue extensibility and provides limited afferent input, whereas MET requires the person to initiate and sustain a controlled isometric contraction against a counterforce. This active engagement generates precise proprioceptive and efferent feedback, which can enhance motor learning, strengthen cortical sensorimotor representation of the affected limb, and promote use-dependent neuroplasticity (10). From a neurophysiological standpoint, combining voluntary effort with precise joint positioning may more effectively activate descending corticospinal pathways and improve the integration of sensory and motor signals, both of which are critical for motor recovery after stroke (45).

Unlike pharmacological interventions such as botulinum toxin, which act directly at the neuromuscular junction to reduce spasticity without requiring person participation, MET depends on active involvement from the person. Effective application of MET relies on several person-related factors: the ability to understand and follow instructions, the capacity to initiate and sustain a controlled isometric contraction, and sufficient residual motor function to generate a meaningful effort. For this reason, stroke survivors with significant cognitive impairment, aphasia, or severe motor paralysis may not be suitable candidates for MET, or may need substantial modification of the technique. This distinction matters clinically: while MET can be a valuable adjunct for individuals with preserved cognitive and motor function, it is not universally applicable across all recovery phases or person subgroups. Person selection should therefore be guided by careful assessment of cognitive status, motor function, and overall rehabilitation goals.

Despite growing interest in MET and promising preclinical findings, translating it into evidence-based practice for PSS presents both methodological and conceptual challenges. One major issue is the lack of high-quality clinical trials. Existing studies tend to have small sample sizes, short follow-up periods, and a narrow focus on impairment-level outcomes such as localized tenderness and range of motion, while often overlooking meaningful changes in activity, participation, or pain-related interference (46). This makes it difficult to draw firm conclusions about the true clinical efficacy of MET.

A further limitation lies in the evidence supporting the proposed mechanisms of MET. Much of what we know about these mechanisms comes from a small number of experimental studies, most of which were conducted in asymptomatic individuals or non-stroke populations (30, 31), and often relied on surrogate measures such as H-reflex amplitude. While these measures reflect spinal excitability, they are not direct indicators of clinical spasticity reduction. These findings offer useful insights into how MET might work, but they need to be interpreted with caution. Neurophysiological effects observed in controlled experimental settings do not always translate into clinical benefit, and the current evidence does not yet support strong mechanistic claims. This gap points to the need for further research that directly links MET-induced neurophysiological changes to meaningful clinical outcomes in stroke survivors.

Moving from promising but still preliminary evidence toward established clinical practice will require a coordinated research agenda. This agenda needs to address current limitations, including small sample sizes, heterogeneous protocols, and a lack of direct evidence, while also using emerging technologies to expand the applicability of MET.

First, more rigorous comparative-effectiveness research is needed. Current studies have small sample sizes, short follow-up periods, and focus mainly on impairment-level outcomes such as muscle tone and range of motion, making firm conclusions about MET’s clinical efficacy difficult (47). Future trials should be adequately powered to compare MET not only with standard care or placebo, but also with other manual and exercise-based interventions like joint mobilization, soft tissue mobilization, and proprioceptive neuromuscular facilitation, to clarify its specific value. As MET may work best in a multi-modal approach, research should also explore combining it with adjunctive therapies such as biofeedback, isokinetic training, and blood flow restriction training to evaluate synergistic effects on motor function and neuroplasticity.

Second, procedural standardization is essential for replicable research and consistent outcomes. Currently, MET application varies widely across practitioners in contraction duration, intensity, repetition, and technique variant (e.g., autogenic vs. reciprocal inhibition). This variation makes it difficult to compare results across studies and to build a coherent evidence base. A key priority is therefore to develop and validate consensus-based treatment protocols, along with practitioner certification standards to ensure consistent application.

Third, technology could help address MET’s dependence on active participation, which limits its use in stroke survivors with cognitive or motor deficits. AI-assisted wearable sensors could provide real-time biofeedback to guide precise contractions, allowing individuals with limited control to participate. More ambitiously, brain-computer interfaces that decode motor intention and translate it into MET-guided robotic movement could enable task-specific training even in patients with severe weakness, connecting cortical intent to motor execution.

Last, phase-specific frameworks should align MET with changing needs during stroke recovery. In the acute phase, MET could combine with cryotherapy to modulate tone and reduce nociceptive input. In the subacute phase, when neural plasticity peaks, MET can pair with neuromodulation (e.g., transcranial direct current stimulation or botulinum toxin) to support motor relearning. In the chronic phase, MET can integrate with advanced motor learning approaches, such as constraint-induced movement therapy or task-specific training, to maximize functional gains and prevent secondary complications.

Taken together, strengthening the evidence base for MET in PSS will need to focus on comparative effectiveness research, procedural standardization, technological innovation, and phase-specific application. A framework built around these priorities will be key to establishing MET as a practical and effective adjunctive intervention in stroke rehabilitation.

## 6 Conclusion

The MET is a promising non-invasive intervention for PSS. What early evidence we have suggests MET may work through spinal and supraspinal mechanisms to reduce spasticity and relieve pain, though support for these mechanisms remains limited. Initial clinical findings show improvements in muscle tone, range of motion, and functional outcomes, but these results need to be viewed with caution given the small number of direct studies and the variability in treatment protocols. Furthermore, its reliance on active participation means it may not be suitable for all stroke survivors, and the absence of standardized protocols limits its broader use in clinical practice. Future work should focus on high-quality trials, procedural standardization, and technological innovation to help integrate MET into evidence-based stroke rehabilitation.
